# 2,9-Dichloro-1,10-phenanthroline

**DOI:** 10.1107/S1600536809011180

**Published:** 2009-03-31

**Authors:** Said Nadeem, Muhammad Raza Shah, Seik Weng Ng

**Affiliations:** a HEJ Research Institute of Chemistry, International Center for Chemical and Biological Sciences, University of Karachi, Karachi 75270, Pakistan; b Department of Chemistry, University of Malaya, 50603 Kuala Lumpur, Malaysia

## Abstract

The title mol­ecule, C_12_H_6_Cl_2_N_2_, is almost planar (the r.m.s. deviation of C atoms is 0.04 Å). The C—N and C—C distances indicate delocalization of the π-electrons in the aromatic fused-ring system.

## Related literature

For the synthesis, see: Yamada *et al.* (1990 ▶). The compound is used for the synthesis of other phenanthroline-like heterocycles; see: Hamilton *et al.* (2004 ▶); Ohira *et al.* (2005 ▶); Zong & Thummel (2004 ▶, 2005 ▶).
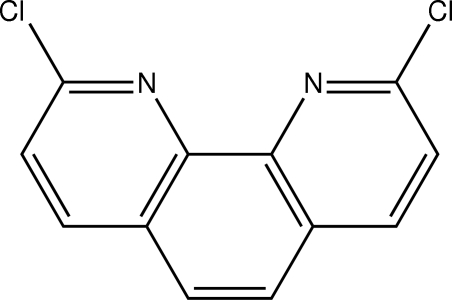


## Experimental

### 

#### Crystal data

C_12_H_6_Cl_2_N_2_
*M* *_r_* = 249.09
Orthorhombic, 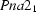

*a* = 19.4035 (3) Å
*b* = 4.4330 (1) Å
*c* = 11.7695 (2) Å
*V* = 1012.36 (3) Å^3^
*Z* = 4
Mo *K*α radiation
μ = 0.61 mm^−1^
*T* = 100 K
0.36 × 0.18 × 0.02 mm

#### Data collection

Bruker SMART APEX diffractometer
Absorption correction: multi-scan (*SADABS*; Sheldrick, 1996 ▶) *T*_min_ = 0.811, *T*_max_ = 0.988
8646 measured reflections
2315 independent reflections
2248 reflections with *I* > 2σ(*I*)
*R*_int_ = 0.022

#### Refinement

*R*[*F* ^2^ > 2σ(*F* ^2^)] = 0.022
*wR*(*F* ^2^) = 0.061
*S* = 1.02
2315 reflections
145 parameters
1 restraint
H-atom parameters constrained
Δρ_max_ = 0.27 e Å^−3^
Δρ_min_ = −0.16 e Å^−3^
Absolute structure: Flack (1983 ▶), 1097 Friedel pairs
Flack parameter: −0.01 (4)

### 

Data collection: *APEX2* (Bruker, 2008 ▶); cell refinement: *SAINT* (Bruker, 2008 ▶); data reduction: *SAINT*; program(s) used to solve structure: *SHELXS97* (Sheldrick, 2008 ▶); program(s) used to refine structure: *SHELXL97* (Sheldrick, 2008 ▶); molecular graphics: *X-SEED* (Barbour, 2001 ▶); software used to prepare material for publication: *publCIF* (Westrip, 2009 ▶).

## Supplementary Material

Crystal structure: contains datablocks global, I. DOI: 10.1107/S1600536809011180/tk2404sup1.cif

Structure factors: contains datablocks I. DOI: 10.1107/S1600536809011180/tk2404Isup2.hkl

Additional supplementary materials:  crystallographic information; 3D view; checkCIF report

## Figures and Tables

**Fig. 1.**
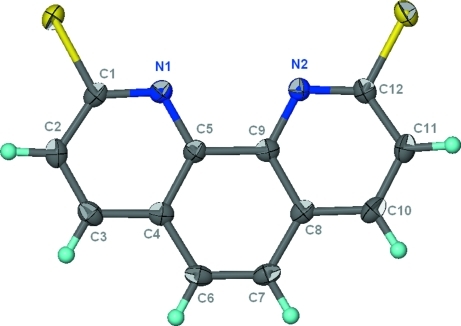
Thermal ellipsoid plot (Barbour, 2001) plot of C12H6Cl2N2 at the 70% probability level; hydrogen atoms are drawn as spheres of arbitrary radius.

## supplementary crystallographic information

### Experimental

A mixture of 6,7-dihydro-3*H*-[1,4]diazepino[1,2,3,4-*lmn*][1,10]phenanthroline-3,9(5*H*)-dione (1.7 g, 6.7 mmol) and phosphorus pentachloride (3 g, 14.4 mmol) was reacted in thionyl chloride (20 ml, 170 mmol) for 16 h at room temperature. The thionyl chloride was removed under reduced pressure and the residue was suspended in cold ammonium hydroxide. A light-tan precipitate was formed which was dissolved in hot benzene; crystals were obtained upon recrystallization from benzene (1.1 g 65%)

### Refinement

Carbon-bound H-atoms were placed in calculated positions (C—H 0.95 Å) and were included in the refinement in the riding model approximation, with *U*(H) set to 1.2*U*_eq_(C).

### Crystal data

**Table:**

C_12_H_6_Cl_2_N_2_	*F*(000) = 504
*M**_r_* = 249.09	*D*_x_ = 1.634 Mg m^−^^3^
Orthorhombic, *P**n**a*2_1_	Mo *K*α radiation, λ = 0.71073 Å
Hall symbol: P 2c -2n	Cell parameters from 5870 reflections
*a* = 19.4035 (3) Å	θ = 2.7–28.3°
*b* = 4.4330 (1) Å	µ = 0.61 mm^−^^1^
*c* = 11.7695 (2) Å	*T* = 100 K
*V* = 1012.36 (3) Å^3^	Plate, colourless
*Z* = 4	0.36 × 0.18 × 0.02 mm

### Data collection

**Table:**

Bruker SMART APEX diffractometer	2315 independent reflections
Radiation source: fine-focus sealed tube	2248 reflections with *I* > 2σ(*I*)
graphite	*R*_int_ = 0.022
ω scans	θ_max_ = 27.5°, θ_min_ = 2.1°
Absorption correction: multi-scan (*SADABS*; Sheldrick, 1996)	*h* = −25→25
*T*_min_ = 0.811, *T*_max_ = 0.988	*k* = −5→5
8646 measured reflections	*l* = −15→15

### Refinement

**Table:**

Refinement on *F*^2^	Secondary atom site location: difference Fourier map
Least-squares matrix: full	Hydrogen site location: inferred from neighbouring sites
*R*[*F*^2^ > 2σ(*F*^2^)] = 0.022	H-atom parameters constrained
*wR*(*F*^2^) = 0.061	*w* = 1/[σ^2^(*F*_o_^2^) + (0.0436*P*)^2^ + 0.147*P*] where *P* = (*F*_o_^2^ + 2*F*_c_^2^)/3
*S* = 1.02	(Δ/σ)_max_ = 0.001
2315 reflections	Δρ_max_ = 0.27 e Å^−^^3^
145 parameters	Δρ_min_ = −0.16 e Å^−^^3^
1 restraint	Absolute structure: Flack (1983), 1097 Friedel pairs
Primary atom site location: structure-invariant direct methods	Flack parameter: −0.01 (4)

### Fractional atomic coordinates and isotropic or equivalent isotropic displacement parameters (Å^2^)

**Table:**

	*x*	*y*	*z*	*U*_iso_*/*U*_eq_	
Cl1	0.027691 (18)	0.55270 (8)	0.25010 (3)	0.01876 (10)	
Cl2	0.35805 (2)	0.89704 (8)	0.45699 (4)	0.02052 (10)	
N1	0.11803 (7)	0.4349 (3)	0.40752 (11)	0.0151 (3)	
N2	0.24727 (7)	0.5756 (3)	0.48930 (11)	0.0153 (3)	
C1	0.05604 (8)	0.3672 (3)	0.37354 (12)	0.0158 (3)	
C2	0.01038 (8)	0.1645 (4)	0.42492 (13)	0.0183 (3)	
H2	−0.0341	0.1269	0.3945	0.022*	
C3	0.03326 (8)	0.0226 (4)	0.52172 (14)	0.0184 (3)	
H3	0.0046	−0.1192	0.5595	0.022*	
C4	0.09941 (8)	0.0884 (3)	0.56464 (14)	0.0159 (3)	
C5	0.14049 (8)	0.2979 (4)	0.50487 (12)	0.0150 (3)	
C6	0.12494 (9)	−0.0487 (3)	0.66693 (14)	0.0178 (3)	
H6	0.0980	−0.1968	0.7048	0.021*	
C7	0.18689 (8)	0.0307 (4)	0.70984 (13)	0.0176 (3)	
H7	0.2025	−0.0587	0.7785	0.021*	
C8	0.22933 (8)	0.2478 (3)	0.65313 (13)	0.0158 (3)	
C9	0.20767 (8)	0.3756 (3)	0.54864 (13)	0.0150 (3)	
C10	0.29315 (8)	0.3417 (4)	0.69870 (14)	0.0189 (3)	
H10	0.3088	0.2647	0.7695	0.023*	
C11	0.33217 (9)	0.5458 (4)	0.63927 (14)	0.0195 (3)	
H11	0.3752	0.6152	0.6676	0.023*	
C12	0.30611 (7)	0.6483 (3)	0.53484 (13)	0.0164 (3)	

### Atomic displacement parameters (Å^2^)

**Table:**

	*U*^11^	*U*^22^	*U*^33^	*U*^12^	*U*^13^	*U*^23^
Cl1	0.01835 (17)	0.02130 (17)	0.01663 (17)	0.00087 (13)	−0.00441 (14)	−0.00032 (15)
Cl2	0.01900 (17)	0.02184 (16)	0.02072 (17)	−0.00573 (14)	0.00151 (15)	−0.00218 (15)
N1	0.0165 (6)	0.0163 (6)	0.0124 (6)	0.0010 (5)	0.0008 (5)	−0.0019 (5)
N2	0.0154 (6)	0.0158 (6)	0.0146 (6)	0.0001 (5)	0.0000 (5)	−0.0014 (4)
C1	0.0173 (7)	0.0174 (7)	0.0126 (7)	0.0046 (6)	−0.0004 (6)	−0.0028 (5)
C2	0.0154 (7)	0.0197 (7)	0.0199 (8)	0.0007 (6)	−0.0002 (6)	−0.0062 (6)
C3	0.0183 (8)	0.0186 (7)	0.0182 (8)	−0.0024 (6)	0.0051 (6)	−0.0023 (6)
C4	0.0192 (7)	0.0150 (7)	0.0136 (7)	0.0014 (5)	0.0028 (6)	−0.0030 (5)
C5	0.0158 (7)	0.0170 (7)	0.0121 (6)	0.0017 (5)	0.0013 (5)	−0.0018 (6)
C6	0.0220 (8)	0.0167 (7)	0.0146 (7)	0.0000 (6)	0.0060 (7)	0.0008 (6)
C7	0.0231 (8)	0.0177 (7)	0.0122 (6)	0.0047 (6)	0.0017 (6)	0.0004 (6)
C8	0.0181 (7)	0.0161 (7)	0.0132 (7)	0.0036 (6)	0.0001 (6)	−0.0022 (5)
C9	0.0178 (7)	0.0143 (6)	0.0130 (7)	0.0015 (5)	0.0014 (6)	−0.0027 (6)
C10	0.0212 (8)	0.0212 (8)	0.0145 (7)	0.0056 (6)	−0.0033 (6)	−0.0020 (6)
C11	0.0174 (8)	0.0223 (8)	0.0187 (8)	0.0003 (6)	−0.0041 (6)	−0.0052 (6)
C12	0.0156 (7)	0.0170 (7)	0.0166 (7)	−0.0003 (6)	0.0022 (6)	−0.0030 (6)

### Geometric parameters (Å, °)

**Table:**

Cl1—C1	1.758 (2)	C4—C6	1.437 (2)
Cl2—C12	1.752 (2)	C5—C9	1.443 (2)
N1—C1	1.303 (2)	C6—C7	1.350 (2)
N1—C5	1.368 (2)	C6—H6	0.9500
N2—C12	1.302 (2)	C7—C8	1.432 (2)
N2—C9	1.365 (2)	C7—H7	0.9500
C1—C2	1.399 (2)	C8—C10	1.412 (2)
C2—C3	1.375 (2)	C8—C9	1.418 (2)
C2—H2	0.9500	C10—C11	1.372 (2)
C3—C4	1.410 (2)	C10—H10	0.9500
C3—H3	0.9500	C11—C12	1.405 (2)
C4—C5	1.412 (2)	C11—H11	0.9500
			
C1—N1—C5	116.68 (13)	C4—C6—H6	119.7
C12—N2—C9	116.36 (14)	C6—C7—C8	120.86 (14)
N1—C1—C2	126.86 (14)	C6—C7—H7	119.6
N1—C1—Cl1	115.78 (12)	C8—C7—H7	119.6
C2—C1—Cl1	117.36 (12)	C10—C8—C9	118.15 (14)
C3—C2—C1	116.59 (14)	C10—C8—C7	121.70 (14)
C3—C2—H2	121.7	C9—C8—C7	120.16 (14)
C1—C2—H2	121.7	N2—C9—C8	122.43 (14)
C2—C3—C4	119.74 (15)	N2—C9—C5	118.74 (14)
C2—C3—H3	120.1	C8—C9—C5	118.81 (14)
C4—C3—H3	120.1	C11—C10—C8	118.99 (15)
C3—C4—C5	118.14 (15)	C11—C10—H10	120.5
C3—C4—C6	121.77 (15)	C8—C10—H10	120.5
C5—C4—C6	120.08 (14)	C10—C11—C12	117.45 (15)
N1—C5—C4	121.97 (14)	C10—C11—H11	121.3
N1—C5—C9	118.72 (14)	C12—C11—H11	121.3
C4—C5—C9	119.29 (14)	N2—C12—C11	126.57 (15)
C7—C6—C4	120.66 (15)	N2—C12—Cl2	116.43 (12)
C7—C6—H6	119.7	C11—C12—Cl2	117.00 (12)
			
C5—N1—C1—C2	−1.3 (2)	C12—N2—C9—C8	−1.2 (2)
C5—N1—C1—Cl1	178.60 (10)	C12—N2—C9—C5	177.09 (13)
N1—C1—C2—C3	0.2 (2)	C10—C8—C9—N2	2.5 (2)
Cl1—C1—C2—C3	−179.66 (12)	C7—C8—C9—N2	−177.70 (13)
C1—C2—C3—C4	0.8 (2)	C10—C8—C9—C5	−175.77 (13)
C2—C3—C4—C5	−0.8 (2)	C7—C8—C9—C5	4.0 (2)
C2—C3—C4—C6	178.52 (14)	N1—C5—C9—N2	−2.1 (2)
C1—N1—C5—C4	1.3 (2)	C4—C5—C9—N2	179.17 (13)
C1—N1—C5—C9	−177.33 (14)	N1—C5—C9—C8	176.22 (13)
C3—C4—C5—N1	−0.4 (2)	C4—C5—C9—C8	−2.5 (2)
C6—C4—C5—N1	−179.63 (14)	C9—C8—C10—C11	−1.5 (2)
C3—C4—C5—C9	178.30 (14)	C7—C8—C10—C11	178.68 (15)
C6—C4—C5—C9	−1.0 (2)	C8—C10—C11—C12	−0.5 (2)
C3—C4—C6—C7	−176.22 (15)	C9—N2—C12—C11	−1.1 (2)
C5—C4—C6—C7	3.0 (2)	C9—N2—C12—Cl2	178.34 (10)
C4—C6—C7—C8	−1.5 (2)	C10—C11—C12—N2	2.0 (2)
C6—C7—C8—C10	177.72 (15)	C10—C11—C12—Cl2	−177.45 (12)
C6—C7—C8—C9	−2.1 (2)		
